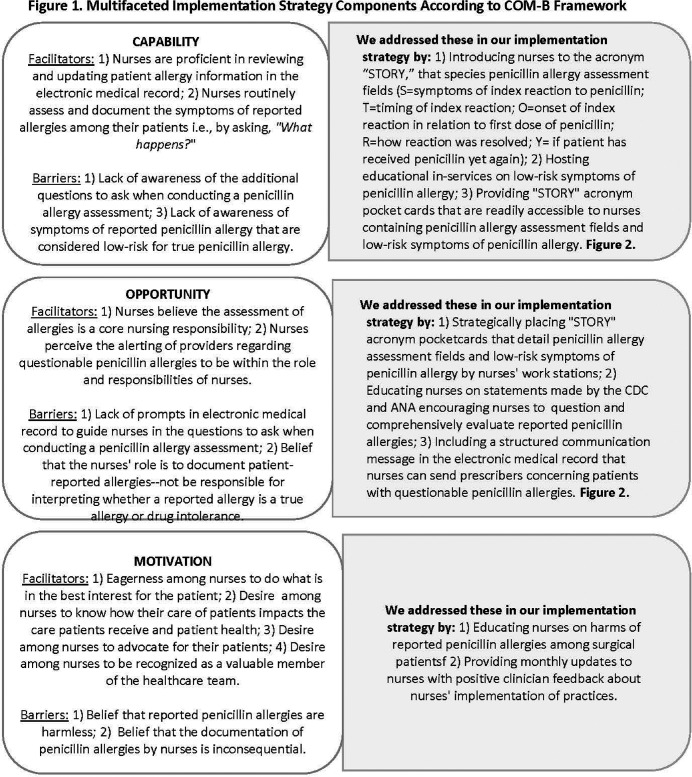# Multifaceted Implementation Strategy to Improve the Comprehensive Assessment of Penicillin Allergies in Perioperative Patient

**DOI:** 10.1017/ash.2024.100

**Published:** 2024-09-16

**Authors:** Eileen Carter, Carol Schramm, Katherine Zavez, Meagan Zolla, Katelyn Baron, David Banach

**Affiliations:** University of Connecticut; UConn Health; Unviersity of Connecticut School of Medicine

## Abstract

**Background:** The CDC recommends that nurses improve the evaluation of penicillin allergies as part of antimicrobial stewardship programs. We evaluated the feasibility of a multifaceted implementation strategy to improve nurses’ documentation of penicillin allergy histories and to encourage nurses to notify prescribers of patients with low-risk symptoms of reported penicillin allergy. The implementation strategy was guided by the COM-B model of behavior change and addressed nurses’ capability, opportunity, and motivation to implement practices (Figure 1). The implementation strategy included education on the STORY mnemonic that details the questions of a penicillin allergy history, education on low-risk symptoms of penicillin allergy, dot phrases in EPIC to facilitate nurses’ documentation of STORY and communication of patients with low-risk penicillin allergy symptoms, and educational pocket cards and flyers. We define feasibility as the implementation and acceptability of practices. **Methods:** This was a six-month feasibility study conducted in an outpatient perioperative area. We compared penicillin allergy documentation pre- and post- implementation strategy and report on nurses’ notification of prescribers regarding patients with low-risk penicillin allergies in the post-implementation period. We engaged nurses in a focus group to assess factors that facilitated or hindered practice adoption. **Results:** A total of 426 unique patients with 482 penicillin allergy records were included in our study (n= 207 records pre-implementation, n = 275 records post-implementation). We found little to no change in the percentage of records that included symptom information post vs. pre-implementation (88.36% vs 88.41%). A greater percentage of allergy records in the post vs. pre-implementation periods included information on: timing/years since reaction (25.6% vs. 8.2%), onset of reaction (20.7% vs. 0%), resolution of symptoms (20.4% vs. 0%), and penicillin re-exposure (21.1% vs. 2.4%). There were 24 documented instances of nurses’ notifying prescribers of patients with a low-risk penicillin allergy. Focus group data revealed nurses perceived their comprehensive documentation of penicillin allergies highly acceptable and likely to improve patient care and outcomes. Whereas nurses’ notification of prescribers concerning patients meeting low-risk penicillin allergy criteria had little appeal. Nurses described the STORY mnemonic, pocket cards describing the penicillin allergy assessment mnemonic, and the associated dot phrase in EPIC as particularly helpful. **Conclusions:** A multifaceted implementation strategy showed promise in improving the comprehensive documentation of penicillin allergy histories. Future studies are needed to determine the efficacy of the multifaceted implementation strategy on penicillin allergy documentation, the selection of antibiotic prophylactic treatment, and clinical outcomes among surgical patients.

**Figure:**